# 2-Amino-4-(2-chloro­phen­yl)-6-(naph­thalen-1-yl)pyridine-3-carbonitrile

**DOI:** 10.1107/S1600536812020909

**Published:** 2012-05-23

**Authors:** Hong-Xia Wei, Jing Zhu, Ming Li, Jian-qiang Wang, Cheng Guo

**Affiliations:** a College of Science, Nanjing University of Technology, Xinmofan Road No. 5 Nanjing, Nanjing 210009, People’s Republic of China; b College of Food Science and Light Industry, Nanjing University of Technology, Xinmofan Road No. 5 Nanjing, Nanjing 210009, People’s Republic of China

## Abstract

In the title compound, C_22_H_14_ClN_3_, prepared by a one-pot reaction under microwave irradiation, the dihedral angles between the central pyridine ring and the pendant naphthyl and chloro­benzene ring systems are 49.2 (2) and 58.2 (3)°, respectively. In the crystal, inversion dimers linked by pairs of N—H⋯N hydrogen bonds generate *R*_2_^2^(8) loops. The pyridine N atom is the acceptor.

## Related literature

For the use of 2-amino-3-cyano­pyridines as inter­mediates in the preparation of heterocyclic compounds, see: Shishoo *et al.* (1983 ▶). For the synthesis, see: Mantri *et al.* (2008 ▶). For related structures, see: Mkhalid *et al.* (2006 ▶).
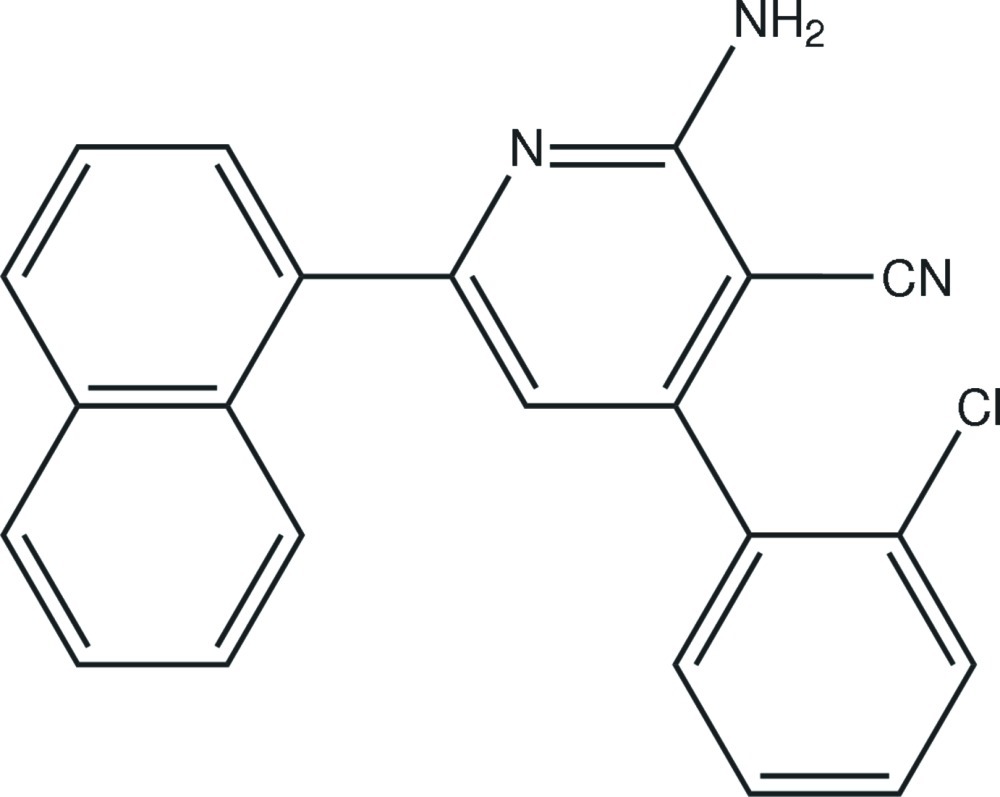


## Experimental

### 

#### Crystal data

C_22_H_14_ClN_3_
*M* *_r_* = 355.81
Monoclinic, 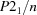

*a* = 12.275 (3) Å
*b* = 4.6490 (9) Å
*c* = 30.887 (6) Å
β = 90.18 (3)°
*V* = 1762.6 (6) Å^3^
*Z* = 4
Mo *K*α radiation
μ = 0.23 mm^−1^
*T* = 293 K
0.20 × 0.10 × 0.10 mm

#### Data collection

Enraf–Nonius CAD-4 diffractometer
Absorption correction: ψ scan (North *et al.*, 1968 ▶) *T*_min_ = 0.956, *T*_max_ = 0.978
3397 measured reflections
3236 independent reflections
1558 reflections with *I* > 2σ(*I*)
*R*_int_ = 0.057
3 standard reflections every 200 reflections intensity decay: 1%

#### Refinement

*R*[*F* ^2^ > 2σ(*F* ^2^)] = 0.069
*wR*(*F* ^2^) = 0.185
*S* = 1.00
3236 reflections
235 parameters
H-atom parameters constrained
Δρ_max_ = 0.20 e Å^−3^
Δρ_min_ = −0.19 e Å^−3^

### 

Data collection: *CAD-4 EXPRESS* (Enraf–Nonius, 1994 ▶); cell refinement: *CAD-4 EXPRESS*; data reduction: *XCAD4* (Harms & Wocadlo, 1995 ▶); program(s) used to solve structure: *SHELXS97* (Sheldrick, 2008 ▶); program(s) used to refine structure: *SHELXL97* (Sheldrick, 2008 ▶); molecular graphics: *SHELXTL* (Sheldrick, 2008 ▶); software used to prepare material for publication: *PLATON* (Spek, 2009 ▶).

## Supplementary Material

Crystal structure: contains datablock(s) global, I. DOI: 10.1107/S1600536812020909/hb6750sup1.cif

Structure factors: contains datablock(s) I. DOI: 10.1107/S1600536812020909/hb6750Isup2.hkl

Supplementary material file. DOI: 10.1107/S1600536812020909/hb6750Isup3.cml

Additional supplementary materials:  crystallographic information; 3D view; checkCIF report

## Figures and Tables

**Table 1:** Hydrogen-bond geometry (Å, °)

*D*—H⋯*A*	*D*—H	H⋯*A*	*D*⋯*A*	*D*—H⋯*A*
N2—H2*A*⋯N1^i^	0.86	2.23	3.086 (5)	176

Symmetry code: (i) 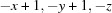.

**Fig. 1.**
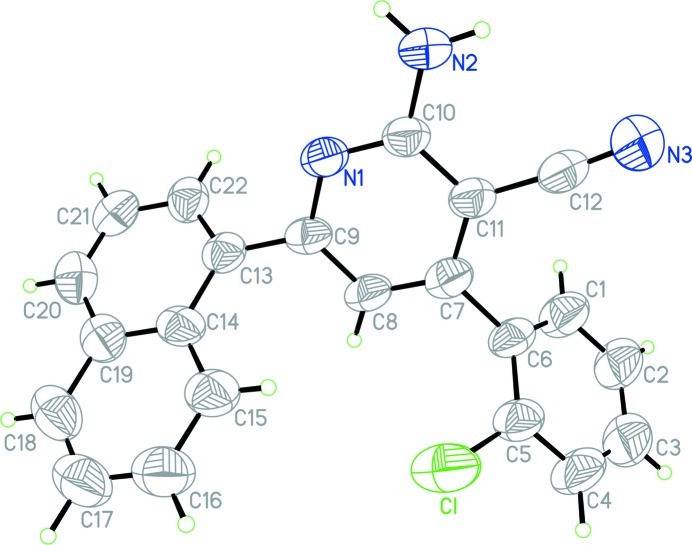
The molecular structure of (I), with displacement ellipsoids drawn at the 50% probability level.

**Fig. 2.**
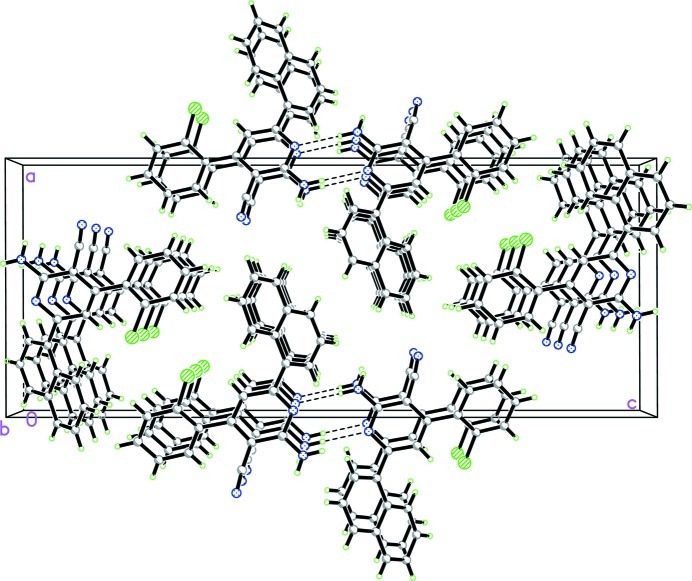
A packing diagram of (I). Hydrogen bonds are shown as dashed lines.

## supplementary crystallographic information

### Comment

The title compound, C_22_H_14_ClN_3_(1), is an intermediate in the synthesis of biologically active molecules (Shishoo *et al.*, 1983; Mantri *et al.* 2008). Herein we report its crystal structure. The molecular structure of (I) is shown in Fig. 1, and the selected geometric parameters are given in Table 1. In the molecules,the naphthyl (C13—C22) and phenyl ring planes (C1—C6) form torsion angles 49.2 and 58.2 °, respectively, with the middle pyridyl ring plane. In the crystal, there are N—H···N hydrogen bonds, which connect the independent molecules into dimers (Fig. 2 and Tab. 1).

### Experimental

For the prepartion of the title compound (1),a mixture of 2-chlorobenzaldehyde (2 mmol), malononitrile (2 mmol), 1-naphthal-dehyde (2 mmol) and ammonium acetate (16 mmol) was refluxed under microwave irradiation (6 min, WF-4000*M* microwave reaction system). After cooling to room temperature, the resulting solid product was filtered off and recrystallized from methanol to give the title compound. Colourless needles were obtained by dissolving the title compound (0.5 g) in methanol (20 ml) and slowly evaporating the solvent at room temperature for a period of about two weeks.

### Refinement

All H atoms were positioned geometrically, with C—H = 0.86 Å and N—H = 0.93 Å for aromatic and amino H, and constrained to ride on their parent atoms,with *U*_iso_(H) = 1.2*U*_eq_(C,*N*).

### Crystal data

**Table:**

C_22_H_14_ClN_3_	*F*(000) = 736
*M**_r_* = 355.81	*D*_x_ = 1.341 Mg m^−^^3^
Monoclinic, *P*2_1_/*n*	Melting point: 421 K
Hall symbol: -P 2yn	Mo *K*α radiation, λ = 0.71073 Å
*a* = 12.275 (3) Å	Cell parameters from 25 reflections
*b* = 4.6490 (9) Å	θ = 9–12°
*c* = 30.887 (6) Å	µ = 0.23 mm^−^^1^
β = 90.18 (3)°	*T* = 293 K
*V* = 1762.6 (6) Å^3^	Needle, colourless
*Z* = 4	0.20 × 0.10 × 0.10 mm

### Data collection

**Table:**

Enraf–Nonius CAD-4 diffractometer	1558 reflections with *I* > 2σ(*I*)
Radiation source: fine-focus sealed tube	*R*_int_ = 0.057
Graphite monochromator	θ_max_ = 25.5°, θ_min_ = 1.3°
ω/2θ scans	*h* = −14→0
Absorption correction: ψ scan (North *et al.*, 1968)	*k* = 0→5
*T*_min_ = 0.956, *T*_max_ = 0.978	*l* = −37→37
3397 measured reflections	3 standard reflections every 200 reflections
3236 independent reflections	intensity decay: 1%

### Refinement

**Table:**

Refinement on *F*^2^	Primary atom site location: structure-invariant direct methods
Least-squares matrix: full	Secondary atom site location: difference Fourier map
*R*[*F*^2^ > 2σ(*F*^2^)] = 0.069	Hydrogen site location: inferred from neighbouring sites
*wR*(*F*^2^) = 0.185	H-atom parameters constrained
*S* = 1.00	*w* = 1/[σ^2^(*F*_o_^2^) + (0.050*P*)^2^ + 1.9*P*] where *P* = (*F*_o_^2^ + 2*F*_c_^2^)/3
3236 reflections	(Δ/σ)_max_ < 0.001
235 parameters	Δρ_max_ = 0.20 e Å^−^^3^
0 restraints	Δρ_min_ = −0.19 e Å^−^^3^

### Special details

**Table:**

Geometry. All e.s.d.'s (except the e.s.d. in the dihedral angle between two l.s. planes) are estimated using the full covariance matrix. The cell e.s.d.'s are taken into account individually in the estimation of e.s.d.'s in distances, angles and torsion angles; correlations between e.s.d.'s in cell parameters are only used when they are defined by crystal symmetry. An approximate (isotropic) treatment of cell e.s.d.'s is used for estimating e.s.d.'s involving l.s. planes.
Refinement. Refinement of *F*^2^ against ALL reflections. The weighted *R*-factor *wR* and goodness of fit *S* are based on *F*^2^, conventional *R*-factors *R* are based on *F*, with *F* set to zero for negative *F*^2^. The threshold expression of *F*^2^ > σ(*F*^2^) is used only for calculating *R*-factors(gt) *etc*. and is not relevant to the choice of reflections for refinement. *R*-factors based on *F*^2^ are statistically about twice as large as those based on *F*, and *R*- factors based on ALL data will be even larger.

### Fractional atomic coordinates and isotropic or equivalent isotropic displacement parameters (Å^2^)

**Table:**

	*x*	*y*	*z*	*U*_iso_*/*U*_eq_	
Cl	0.31710 (12)	0.7045 (4)	0.20602 (4)	0.0872 (5)	
N1	0.4498 (3)	0.3780 (9)	0.05627 (11)	0.0550 (10)	
C1	0.6080 (4)	0.3185 (13)	0.20923 (15)	0.0773 (16)	
H1B	0.6571	0.2335	0.1903	0.093*	
N2	0.6054 (3)	0.6314 (10)	0.04127 (11)	0.0763 (14)	
H2A	0.5897	0.6189	0.0142	0.092*	
H2B	0.6635	0.7198	0.0494	0.092*	
C2	0.6330 (5)	0.3305 (14)	0.25299 (16)	0.0863 (18)	
H2C	0.6981	0.2532	0.2632	0.104*	
N3	0.7314 (4)	0.8313 (12)	0.13555 (14)	0.0968 (17)	
C3	0.5613 (5)	0.4568 (15)	0.28131 (17)	0.090 (2)	
H3A	0.5779	0.4680	0.3107	0.109*	
C4	0.4654 (5)	0.5656 (13)	0.26593 (15)	0.0814 (17)	
H4A	0.4162	0.6475	0.2851	0.098*	
C5	0.4402 (4)	0.5563 (11)	0.22255 (14)	0.0634 (13)	
C6	0.5115 (4)	0.4305 (11)	0.19312 (13)	0.0578 (13)	
C7	0.4887 (4)	0.4158 (11)	0.14580 (14)	0.0579 (13)	
C8	0.3990 (4)	0.2782 (11)	0.12958 (13)	0.0597 (13)	
H8A	0.3499	0.1926	0.1485	0.072*	
C9	0.3800 (3)	0.2645 (10)	0.08536 (13)	0.0507 (11)	
C10	0.5383 (4)	0.5115 (11)	0.07123 (14)	0.0542 (12)	
C11	0.5606 (3)	0.5421 (11)	0.11589 (14)	0.0568 (12)	
C12	0.6549 (4)	0.7022 (13)	0.12856 (14)	0.0673 (15)	
C13	0.2812 (4)	0.1290 (10)	0.06732 (14)	0.0533 (12)	
C14	0.1748 (4)	0.1886 (11)	0.08349 (14)	0.0577 (13)	
C15	0.1539 (4)	0.3973 (12)	0.11570 (15)	0.0682 (14)	
H15A	0.2113	0.5015	0.1276	0.082*	
C16	0.0499 (5)	0.4471 (14)	0.12953 (18)	0.0842 (17)	
H16A	0.0379	0.5848	0.1508	0.101*	
C17	−0.0383 (5)	0.2979 (16)	0.11261 (19)	0.090 (2)	
H17A	−0.1083	0.3344	0.1226	0.108*	
C18	−0.0217 (4)	0.0997 (14)	0.08151 (19)	0.0807 (17)	
H18A	−0.0808	−0.0007	0.0702	0.097*	
C19	0.0835 (4)	0.0418 (12)	0.06576 (16)	0.0629 (13)	
C20	0.1015 (4)	−0.1567 (12)	0.03181 (17)	0.0735 (15)	
H20A	0.0425	−0.2559	0.0202	0.088*	
C21	0.2025 (4)	−0.2061 (11)	0.01577 (15)	0.0679 (14)	
H21A	0.2118	−0.3334	−0.0071	0.082*	
C22	0.2927 (4)	−0.0649 (11)	0.03379 (15)	0.0613 (13)	
H22A	0.3618	−0.1027	0.0229	0.074*	

### Atomic displacement parameters (Å^2^)

**Table:**

	*U*^11^	*U*^22^	*U*^33^	*U*^12^	*U*^13^	*U*^23^
Cl	0.0919 (10)	0.1025 (12)	0.0674 (9)	0.0187 (9)	0.0206 (7)	−0.0003 (8)
N1	0.057 (2)	0.066 (3)	0.042 (2)	0.005 (2)	0.0057 (17)	0.003 (2)
C1	0.086 (4)	0.095 (4)	0.051 (3)	0.012 (3)	−0.004 (3)	−0.004 (3)
N2	0.065 (3)	0.118 (4)	0.046 (2)	−0.017 (3)	0.0128 (19)	−0.002 (3)
C2	0.100 (4)	0.106 (5)	0.052 (3)	−0.009 (4)	−0.012 (3)	0.006 (4)
N3	0.075 (3)	0.137 (5)	0.079 (3)	−0.029 (3)	0.009 (3)	−0.024 (3)
C3	0.100 (5)	0.123 (6)	0.049 (3)	−0.024 (4)	−0.007 (3)	0.008 (4)
C4	0.105 (4)	0.100 (5)	0.039 (3)	−0.011 (4)	0.013 (3)	−0.007 (3)
C5	0.073 (3)	0.072 (4)	0.045 (3)	−0.006 (3)	0.011 (2)	0.002 (3)
C6	0.073 (3)	0.064 (3)	0.036 (2)	−0.002 (3)	0.006 (2)	0.001 (2)
C7	0.064 (3)	0.066 (3)	0.043 (3)	0.009 (3)	0.003 (2)	0.000 (2)
C8	0.069 (3)	0.072 (3)	0.038 (2)	−0.004 (3)	0.009 (2)	0.002 (3)
C9	0.057 (3)	0.053 (3)	0.042 (2)	0.006 (2)	0.009 (2)	0.008 (2)
C10	0.050 (3)	0.069 (3)	0.044 (3)	0.006 (3)	0.007 (2)	0.000 (2)
C11	0.051 (3)	0.074 (3)	0.046 (3)	0.006 (3)	0.006 (2)	−0.008 (3)
C12	0.066 (3)	0.094 (4)	0.041 (3)	−0.001 (3)	0.007 (2)	−0.012 (3)
C13	0.060 (3)	0.057 (3)	0.042 (2)	0.004 (3)	0.000 (2)	0.011 (2)
C14	0.057 (3)	0.066 (3)	0.050 (3)	0.005 (3)	0.011 (2)	0.021 (3)
C15	0.069 (3)	0.080 (4)	0.056 (3)	0.003 (3)	0.010 (2)	0.015 (3)
C16	0.083 (4)	0.100 (5)	0.069 (4)	0.020 (4)	0.016 (3)	0.009 (3)
C17	0.067 (4)	0.121 (6)	0.083 (4)	0.021 (4)	0.019 (3)	0.031 (4)
C18	0.058 (3)	0.092 (5)	0.092 (4)	0.000 (3)	0.004 (3)	0.027 (4)
C19	0.062 (3)	0.062 (3)	0.064 (3)	−0.001 (3)	−0.001 (2)	0.018 (3)
C20	0.070 (3)	0.071 (4)	0.080 (4)	−0.001 (3)	−0.014 (3)	0.007 (3)
C21	0.090 (4)	0.063 (3)	0.051 (3)	0.007 (3)	−0.004 (3)	−0.003 (3)
C22	0.063 (3)	0.063 (3)	0.058 (3)	0.001 (3)	−0.007 (2)	0.007 (3)

### Geometric parameters (Å, º)

**Table:**

Cl—C5	1.736 (5)	C9—C13	1.475 (6)
N1—C10	1.332 (5)	C10—C11	1.413 (6)
N1—C9	1.351 (5)	C11—C12	1.429 (7)
C1—C6	1.385 (6)	C13—C22	1.380 (6)
C1—C2	1.386 (6)	C13—C14	1.427 (6)
C1—H1B	0.9300	C14—C15	1.414 (7)
N2—C10	1.360 (5)	C14—C19	1.421 (6)
N2—H2A	0.8600	C15—C16	1.367 (6)
N2—H2B	0.8600	C15—H15A	0.9300
C2—C3	1.375 (7)	C16—C17	1.386 (8)
C2—H2C	0.9300	C16—H16A	0.9300
N3—C12	1.134 (6)	C17—C18	1.347 (8)
C3—C4	1.365 (7)	C17—H17A	0.9300
C3—H3A	0.9300	C18—C19	1.407 (6)
C4—C5	1.375 (6)	C18—H18A	0.9300
C4—H4A	0.9300	C19—C20	1.414 (7)
C5—C6	1.392 (6)	C20—C21	1.356 (6)
C6—C7	1.489 (6)	C20—H20A	0.9300
C7—C8	1.367 (6)	C21—C22	1.401 (6)
C7—C11	1.408 (6)	C21—H21A	0.9300
C8—C9	1.386 (5)	C22—H22A	0.9300
C8—H8A	0.9300		
			
C10—N1—C9	118.0 (4)	C7—C11—C10	118.6 (4)
C6—C1—C2	121.4 (5)	C7—C11—C12	123.1 (4)
C6—C1—H1B	119.3	C10—C11—C12	118.3 (4)
C2—C1—H1B	119.3	N3—C12—C11	175.1 (5)
C10—N2—H2A	120.0	C22—C13—C14	119.0 (4)
C10—N2—H2B	120.0	C22—C13—C9	118.5 (4)
H2A—N2—H2B	120.0	C14—C13—C9	122.5 (4)
C3—C2—C1	119.8 (5)	C15—C14—C19	117.1 (4)
C3—C2—H2C	120.1	C15—C14—C13	123.2 (5)
C1—C2—H2C	120.1	C19—C14—C13	119.6 (5)
C4—C3—C2	119.3 (5)	C16—C15—C14	120.5 (5)
C4—C3—H3A	120.3	C16—C15—H15A	119.7
C2—C3—H3A	120.3	C14—C15—H15A	119.7
C3—C4—C5	121.3 (5)	C15—C16—C17	121.7 (6)
C3—C4—H4A	119.4	C15—C16—H16A	119.1
C5—C4—H4A	119.4	C17—C16—H16A	119.1
C4—C5—C6	120.6 (5)	C18—C17—C16	119.4 (5)
C4—C5—Cl	117.8 (4)	C18—C17—H17A	120.3
C6—C5—Cl	121.5 (4)	C16—C17—H17A	120.3
C1—C6—C5	117.5 (4)	C17—C18—C19	121.2 (6)
C1—C6—C7	119.5 (4)	C17—C18—H18A	119.4
C5—C6—C7	122.9 (4)	C19—C18—H18A	119.4
C8—C7—C11	117.4 (4)	C18—C19—C20	121.8 (5)
C8—C7—C6	122.0 (4)	C18—C19—C14	119.9 (5)
C11—C7—C6	120.6 (4)	C20—C19—C14	118.3 (5)
C7—C8—C9	121.0 (4)	C21—C20—C19	121.8 (5)
C7—C8—H8A	119.5	C21—C20—H20A	119.1
C9—C8—H8A	119.5	C19—C20—H20A	119.1
N1—C9—C8	122.2 (4)	C20—C21—C22	119.9 (5)
N1—C9—C13	116.0 (4)	C20—C21—H21A	120.1
C8—C9—C13	121.8 (4)	C22—C21—H21A	120.1
N1—C10—N2	116.7 (4)	C13—C22—C21	121.4 (5)
N1—C10—C11	122.8 (4)	C13—C22—H22A	119.3
N2—C10—C11	120.5 (4)	C21—C22—H22A	119.3
			
C6—C1—C2—C3	−0.3 (9)	N1—C10—C11—C12	−176.8 (5)
C1—C2—C3—C4	0.9 (9)	N2—C10—C11—C12	0.1 (7)
C2—C3—C4—C5	−1.3 (10)	C7—C11—C12—N3	178 (7)
C3—C4—C5—C6	1.1 (9)	C10—C11—C12—N3	−3 (7)
C3—C4—C5—Cl	−179.2 (5)	N1—C9—C13—C22	48.9 (6)
C2—C1—C6—C5	0.1 (8)	C8—C9—C13—C22	−132.1 (5)
C2—C1—C6—C7	179.3 (5)	N1—C9—C13—C14	−131.7 (4)
C4—C5—C6—C1	−0.5 (8)	C8—C9—C13—C14	47.3 (6)
Cl—C5—C6—C1	179.8 (4)	C22—C13—C14—C15	−175.7 (4)
C4—C5—C6—C7	−179.6 (5)	C9—C13—C14—C15	5.0 (7)
Cl—C5—C6—C7	0.7 (7)	C22—C13—C14—C19	1.8 (6)
C1—C6—C7—C8	121.2 (6)	C9—C13—C14—C19	−177.5 (4)
C5—C6—C7—C8	−59.7 (7)	C19—C14—C15—C16	1.6 (7)
C1—C6—C7—C11	−58.1 (7)	C13—C14—C15—C16	179.2 (4)
C5—C6—C7—C11	121.1 (5)	C14—C15—C16—C17	−0.3 (8)
C11—C7—C8—C9	0.0 (7)	C15—C16—C17—C18	−0.5 (9)
C6—C7—C8—C9	−179.3 (4)	C16—C17—C18—C19	−0.2 (9)
C10—N1—C9—C8	−1.3 (7)	C17—C18—C19—C20	−177.2 (5)
C10—N1—C9—C13	177.7 (4)	C17—C18—C19—C14	1.6 (8)
C7—C8—C9—N1	1.8 (7)	C15—C14—C19—C18	−2.2 (7)
C7—C8—C9—C13	−177.1 (5)	C13—C14—C19—C18	−179.9 (4)
C9—N1—C10—N2	−178.0 (4)	C15—C14—C19—C20	176.6 (4)
C9—N1—C10—C11	−1.0 (7)	C13—C14—C19—C20	−1.1 (7)
C8—C7—C11—C10	−2.1 (7)	C18—C19—C20—C21	178.0 (5)
C6—C7—C11—C10	177.1 (4)	C14—C19—C20—C21	−0.8 (7)
C8—C7—C11—C12	177.4 (5)	C19—C20—C21—C22	1.9 (8)
C6—C7—C11—C12	−3.4 (7)	C14—C13—C22—C21	−0.7 (7)
N1—C10—C11—C7	2.7 (7)	C9—C13—C22—C21	178.6 (4)
N2—C10—C11—C7	179.6 (4)	C20—C21—C22—C13	−1.1 (7)

### Hydrogen-bond geometry (Å, º)

**Table:**

*D*—H···*A*	*D*—H	H···*A*	*D*···*A*	*D*—H···*A*
N2—H2*A*···N1^i^	0.86	2.23	3.086 (5)	176

Symmetry code: (i) −*x*+1, −*y*+1, −*z*.
